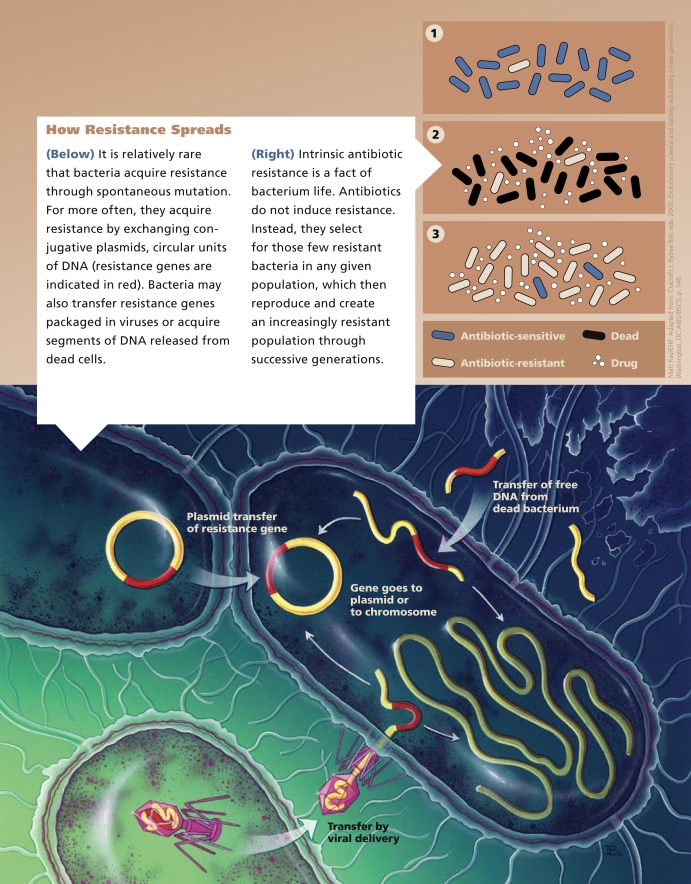# The Landscape of Antibiotic Resistance

**DOI:** 10.1289/ehp.117-a244

**Published:** 2009-06

**Authors:** Noah Rosenblatt-Farrell

In a 1945 interview with *The New York Times*, Alexander Fleming, who won a Nobel Prize that year for his discovery of pencillin, warned that misuse of the drug could result in selection for resistant bacteria. True to this prediction, resistance began to emerge within 10 years of the widescale introduction of penicillin. Indeed, although antibiotics have transformed the medical response to bacterial illness and rendered easily treatable many formerly deadly infections, the mishandling and misprescription of these drugs have transformed the bacterial population such that many antibiotics have partially or entirely lost their efficacy. The problem is severe enough that many experts believe the value of existing antibiotic therapies over the next 100 years is now uncertain. However, some also believe that with a proper response to the current trend in antibiotic resistance, these drugs might once again serve their original function.

## The Cure Is the Catalyst

Antibiotics fight bacteria through a variety of mechanisms. Penicillins, cephalosporins, carbapenems, and vancomycin kill bacteria by damaging or inhibiting the synthesis of bacterial cell walls. Other antibiotics act through effects on bacterial DNA or RNA (quinolones and rifampin), proteins (aminoglycocides, chloramphenicol, tetracyclines, and macrolide antibiotics), or metabolism (trimethoprim and sulfonamides).

Bacteria are said to have “intrinsic resistance” to an antibiotic when their normal characteristics render them immune to the antibiotic’s mechanism of effect. Intrinsic resistance is not affected by misuse of antibiotics. In fact, it is valuable in determining which antibiotic will be most effective against a certain microbe. For example, the outer membrane of gram-negative bacteria makes them relatively impermeable to hydrophobic compounds such as macrolide antibiotics, thus conferring intrinsic resistance to these drugs. Some bacteria can also use temporary strategies in which different genes are expressed or suppressed in order to enable survival in the presence of antibiotics, with expression patterns returning to normal once the threat posed by those particular drugs has passed.

In contrast, bacteria may acquire resistance to an antibiotic by taking on a new characteristic through gene mutation or the transfer of genetic material between bacteria. Acquired characteristics that can make bacteria resistant to an antibiotic include changes to the bacterial membrane that prevent antibiotics from entering the cell. Bacteria may also use enzymes to break down antibiotics, or they may employ “efflux pumps” to remove the antibiotic entirely or reduce its concentration below effective levels.

If a bacterium is able to perform more than one of these functions, it may be resistant to more than one type of antibiotic, resulting in multidrug resistance, according to P.M. Bennett, writing in the March 2008 issue of the *British Journal of Pharmacology*. At the same time, the possession of even a single form of efflux pump can lead to the export of—and protection against—more than one form of antibiotic, thus also conferring multi-drug resistance, adds David McDowell, a professor of food studies at the University of Ulster.

Mutations are relatively rare, occurring in only 1 event per 10^7^–10^10^ bacteria, according to a review by Michael R. Mulvey and Andrew E. Simor in the 17 February 2009 issue of the *Canadian Medical Association Journal*. As an example, Mulvey and Simor pointed to isoniazid resistance among *Mycobacterium tuberculosis*. “This form of resistance is not transferable to other organisms,” they wrote. “The probability of multiple resistance mutations occurring in a single organism is equal to the product of their individual probabilities. This is the rationale behind the use of combination therapy for the management of tuberculosis.”

Of greater concern are “promiscuous” gene transfer systems that allow the sharing of genetic material between bacteria. One genetic transfer strategy is the exchange of conjugative plasmids. These circles of DNA, which are separate from the bacterial chromosome, can replicate independently and move between bacteria carrying antibiotic resistance genes, thereby multiplying antibiotic resistance among successive generations within a bacterial colony. Bacteria may also acquire resistance genes through the spread of transposons or integrons, groups of linked genetic elements.

In the July 2008 issue of the *Journal of Bacteriology*, Michael Gillings and colleagues wrote that class 1 integrons (the most extensively studied type of integron) now appear in 40–70% of gram-negative pathogens in clinical and agricultural samples. “The rapid spread of class 1 integrons through gram-negative and, more recently, into gram-positive species has been facilitated by their location on mobile DNA elements, such as plasmids and transposons, coupled with the selective advantage conferred by their associated antibiotic resistance genes,” they wrote. The authors noted about 10% of sequenced bacterial genomes carry integrons.

In some instances, resistance mechanisms are induced by the presence of an antibiotic, says José L. Martínez, a microbiologist at the Spanish National Center of Biotechnology, but in most cases resistance arises when susceptible bacteria are killed by the antibiotic and only those resistant few prevail and reproduce. In other words, antibiotics don’t cause resistance. Instead, they select for resistant bacteria and increase the proportional prevalence.

Ironically, the impulse to scour equipment and surfaces may sometimes end up worsening this situation, as M. Ann S. McMahon and colleagues pointed out in the January 2007 issue of *Applied and Environmental Microbiology*: “Detergents and solvents have been shown, among other compounds, to induce the [multiple antibiotic resistance] operon,” they wrote, describing food-preservation processes. “This operon regulates the expression of a large number of genes, including those coding for at least one broad-specificity efflux pump (the arcAB efflux pump), which are more strongly expressed under conditions of environmental stress. This suggests a direct linkage between environmental stresses, such as those occurring in foods and the domestic environment, efflux pump expression, and the development of antibiotic resistance.” The authors suggest that the increased use of sublethal bacteriostatic food preservation methods (as opposed to bactericidal methods) may be contributing to antibiotic resistance among food-related pathogens.

## Case in Point

The problem of antibiotic resistance has become widely known in large part because of the emergence of methicillin-resistant *Staphylococcus aureus* (MRSA), an increasingly common bacterial agent with frightening consequences. Initially, most MRSA infections were contracted by hospital in-patients suffering from other underlying conditions. Such infections were dubbed hospital-acquired MRSA (sometimes called healthcare-associated MRSA, and abbreviated in both cases as HA-MRSA). In 1974, 2% of all *S. aureus* infections in the United States were HA-MRSA, according to the Centers for Disease Control and Prevention. By 1995 this figure rose to 22% and by 2004 had reached 64%. More recently, MRSA infections have been reported among otherwise apparently healthy members of the general population who have not undergone hospitalization or any invasive medical procedure within the past year. These infections are known as community-acquired MRSA (CA-MRSA).

In the September 2008 issue of the *Journal of Clinical Microbiology* Fred C. Tenover and colleagues from the CDC reported on an extended study undertaken to characterize MRSA isolates collected as part of the National Health Examination and Nutrition Survey between 2001 and 2004. A total of 19,412 nasal samples had been collected from noninstitutionalized individuals. Between 2001–2002 and 2003–2004, the incidence of *S. aureus* in nasal samples decreased. However, during the same period, the prevalence of MRSA increased, reaching 1.5%.

Moreover, colonization with MRSA can persist even after several years. In a study reported in the 1 April 2009 issue of *Clinical Infectious Diseases*, Ari Robicsek and colleagues examined 1,564 patients after positive MRSA identification and then retested them over a 4-year period. After one year, 48.8% of the patients were still colonized with MRSA. After four years, 21.2% were still colonized. The lesson to be learned, according to the authors, is that “even in the fourth year after a positive clinical culture result, the risk of MRSA colonization does not subside to that of the general patient population.”

Risk factors for acquiring HA-MRSA include recent hospitalization, outpatient visits to the hospital, and nursing home admission. CA-MRSA infections are also associated with antibiotic exposure, chronic illness, injection drug use, athletics (particularly contact sports such as wrestling or those that involve handling a communal object such as a volleyball), or close contact with someone who has one of these characteristics or exposures. Any sharing of equipment, clothing, or athletic facilities, or skin-to-skin contact, also increases the likelihood of acquiring MRSA. However, HA-MRSA and CA-MRSA have started to blend, with traditional risk factors predicting infection less accurately.

## Spotted in the Wild

In the 2001 report *Hogging It! Estimates of Antimicrobial Abuse in Livestock*, the Union of Concerned Scientists estimated that 70% of all antibiotics used in the United States—more than 24 million pounds per year—is routinely put in the food and water of healthy livestock. Antibiotics are used in feed animals to not only control disease but also improve metabolism and reduce dietary requirements by stimulating the growth of microbes that produce vitamins and amino acids. In a review in the May 2007 issue of *EHP*, Amy R. Sapkota and colleagues wrote that the practice of using antibiotics at non-therapeutic levels “has been shown to select for antibiotic resistance in both commensal and pathogenic bacteria in a) the animals themselves; b) subsequent animal-based food products; and c) water, air, and soil samples collected around large-scale animal feeding operations.”

Veterinary antibiotics often are excreted unchanged. In the April 2001 issue of *Applied and Environmental Microbiology*, for instance, J. C. Chee-Sanford and colleagues reported that up to 75% of tetracycline administered to swine was excreted unaltered. The excreted drugs can persist in the environment, creating an opportunity for resistance selection within exposed bacterial populations.

Animal waste handling practices vary considerably between farms, but generally include “land application,” the spreading of waste on the soil surface as a fertilizer, which can result in contamination of soil and surface or ground water. Many conventional farming operations also use waste lagoons, which provide an alternative route by which birds and insects can pick up antibiotic-resistant bacteria.

A study by Jay P. Graham and colleagues in the 1 April 2009 issue of *Science of the Total Environment* reported that flies collected from the areas surrounding a poultry production facility demonstrated resistance consistent with the types of antibiotics being used there. Graham and colleagues suggested that “the carriage of antibiotic resistant enteric bacteria by flies in the poultry production environment increases the potential for human exposure to drug resistant bacteria.”

And there is evidence that antibiotic-resistant bacteria are traveling far. In the January 2008 issue of *Emerging Infectious Diseases*, Maria Sjöland and colleagues documented an unexpectedly high presence in Arctic wildlife of drug-resistant *Escherichia coli*, which the authors speculate may have been transported by migratory birds. In another recent study, published in the March 2009 issue of *FEMS Microbiology Ecology*, Julie M. Rose and colleagues took 472 bacterial isolates from vertebrates in coastal waters off the northeastern United States, including marine mammals, sharks, and birds, and found that 58% demonstrated resistance to at least one antibiotic, whereas 43% were multidrug-resistant.

In 1996, the National Antibiotic Resistance Monitoring System (NARMS) was formed as a joint effort of the Food and Drug Administration (FDA), the Centers for Disease Control and Prevention, and the U.S. Department of Agriculture to collect data on bacteria present in humans and animals. In 2001, NARMS expanded to include sampling of retail meats collected through random purchases from randomly selected groceries. NARMS first began sampling at locations in 6 states in 2002, then increased to 8 states in 2003, 10 in 2004, and 11 in 2008. The most recent NARMS report, *2006 NARMS Retail Meat Annual Report*, presents some staggering numbers. In tests of chicken breast samples collected between 2002 and 2006, an average of 51.1% tested positive for *Campylobacter*, 11.9% for *Salmonella*, 97.7% for *E. coli*, and 82.6% for *Enterococcus*. In many cases, these bacterial isolates also tested positive for resistance to one or more drugs.

One of the first studies to closely examine the occurrence of MRSA on U.S. farms looked at two swine production systems. As reported by Tara C. Smith and colleagues in the 23 January 2009 edition of *PLoS ONE*, one farm had extremely high levels of the ST398 strain of MRSA in both its animal population (49% overall, with 100% occurrence in animals aged 9–12 weeks) and its workers (64%). Yet, none of the animals or workers in the second farming system had MRSA, which may have to do with the source of the animals. Smith explains, “Because the farms got their animals from different sources, we’re guessing MRSA is moving via importation, bringing in pigs already colonized.”

Pharmaceutical factories, themselves, can be another source of antibiotics entering the environment. As Meghan Hessenauer, an environmental scientist at the U.S. Environmental Protection Agency, points out, guidelines for pharmaceutical manufacturing wastes are geared toward the discharge of chemicals used in the process of manufacturing rather than active pharmaceutical ingredients. This, she says, means “there is no regulation and no limits on antibiotics themselves.”

## Other Environmental Inputs

There are best management practices in place to prevent such industrial releases, says Hessenauer. Nevertheless, drugs are still making their way out of at least some manufacturing plants. In one survey of a wastewater treatment plant that received effluent from a penicillin G production facility, published online 18 February 2009 ahead of print in *Environmental Microbiology*, Dong Li and colleagues demonstrated that, compared with upstream samples, effluent and downstream samples showed significantly high levels of resistance for almost all the antibiotics they tested for.

At the household level, recent studies have found a correlation between the disposal of antibiotics and the emergence of resistance. In research performed by Dean A. Seehusen and John Edwards and described in the November–December 2006 issue of the *Journal of the American Board of Family Medicine*, more than half the patients surveyed had flushed unused or expired pharmaceuticals down the toilet. Only 22.9% reported returning unused medication to a pharmacy, and still fewer had received information from a health care provider about proper medication disposal. In the December 2005 issue of *EHP*, Jonathan Bound and Nikolaos Voulvoulis reported similar numbers from a U.K. study in which only 21.8% of survey respondents returned unused medications to their pharmacies.

As with farm animals, antibiotics may be excreted by humans in their original active form. Up to 80% of amoxicillin, for example, may be excreted unaltered in urine. In the October 2000 issue of *Antimicrobial Agents and Chemotherapy*, for instance, Niels Høiby and colleagues reported that excretion of ceftriaxone and ceftazidime in sweat “may have contributed significantly to the present worldwide selection for and spread of MRSA.” When excreted antibiotics do make their way to sewage treatment plants, they aren’t necessarily removed from the water, nor are antibiotic-resistance bacteria. In the December 2005 issue of *The Journal of General and Applied Microbiology* Xavier Vilanova and Anicet R. Blanch reported finding vancomycin- and erythromycin-resistant bacteria in liquid and dried sludge from a treatment plant.

Researchers are still evaluating how disinfectants and antibacterial products such as handsoap may impact antibiotic resistance. In the April 2003 issue of *Clinical Microbiology Reviews*, Peter Gilbert and Andrew J. McBain wrote, “While the regular application and use of antimicrobial handwashing products have been noted to bring about a change in skin flora, this has not been associated with fluctuations in resistance.” The following year, the Board of the International Scientific Forum on Home Hygiene issued a consensus statement declaring “there is no evidence that biocide use has been a significant factor to date in the development of antibiotic resistance in clinical practice—antibiotic misuse is the most significant causative factor.”

The board noted, however, that “it is important to ensure that biocides are used responsibly as part of a good hygiene routine in the domestic setting in order to avoid the possibility of any impact on antimicrobial resistance in the future.” Indeed, the same holds true for community-scale hygiene. In the June 2004 issue of *Ecotoxicology and Environmental Safety*, Richa Shrivastava reported that suboptimal chlorination of water taken from India’s River Gomti appeared to select for multidrug-resistant *Pseudomonas aeruginosa*, an opportunistic pathogen.

## On the Trail of the Resistance Footprint

David Patrick and James Hutchinson suggested in the 17 February 2009 issue of the *Canadian Medical Association Journal* that a “resistance footprint” can help identify and measure antibiotic hazards. That is, everyone connected with antibiotics through production, prescription, consumption, and disposal should consider their own potential contributions to the problem (their “footprint”) as well as their role in preventing the spread of antibiotic resistance. Says Patrick, “The price for use of a specific course of antibiotics isn’t necessarily suffered by the person who takes them. In addition to potential therapeutic benefits from using antibiotics, there is a contribution to the selective pressure to resistance that affects other people.”

Effective stewardship programs that promote the “resistance footprint” concept must acknowledge and address financial incentives for antibiotic use among farmers, on whom the burden of maintaining their herds rests and for whom financial constraints are often a great concern. Patrick says, “When I speak with food producers about pressure to get rid of antibiotics, they say a complication in North America is that we have a common food market between the United States and Canada, so if one side moves and perceives an economic disadvantage, they worry about putting themselves out of business. We need joint Canadian and U.S. support of agricultural regulations.” If farmers could be shown the longer-term economic benefits of stewardship and “footprint” management, he adds, they might be more inclined to adopt strategies that would limit use of antibiotics and reduce resistance.

So far, the majority of efforts to prevent and reduce antibiotic resistance have occurred in the field of health care, with infectious disease practitioners and researchers leading a call to reduce unnecessary use of antibiotics and adopt other stewardship strategies. Hospitals have implemented stewardship programs that bring interested parties together to identify problem drugs, retrieve historical patient data, and review standing formulary policies in order to develop strategies to manage antimicrobial use and monitor resistance patterns.

In 2007, the Infectious Diseases Society of America and the Society for Health-care Epidemiology of America issued its “Guidelines for Developing an Institutional Program to Enhance Antimicrobial Stewardship.” Some of these guidelines are well validated, such as optimizing antimicrobial dosing based on the individual patient, infectious agent, and site of infection. Others—such as substituting one antibiotic for another—have not yet been validated.

Research to identify new antibiotics for which resistance has not yet developed also is ongoing. However, the high cost of new drug development, combined with the more stringent approval criteria adopted in recent years by the FDA, is prohibitive for many larger pharmaceutical companies. The FDA’s desire to prevent further resistance from emerging also means that a “new compound that makes it through regulatory approval will be put on a restricted list to be used only when other antibiotics have failed, thereby limiting its market,” wrote Julian Davies in volume 8, number 7 (2007) of *EMBO Reports*.

New drug development, says Stuart Levy, a professor of molecular biology and microbiology at Tufts University, may therefore end up in the hands of academia or smaller pharmaceutical companies. “The smaller companies are able to focus on a single organism or a single product, devoting their energies and people to that singular focus,” he explains. “In large companies, particularly when you get to the level of animal studies, you have to wait in a queue in order to do your analysis.”

Meanwhile, reining in antibiotic use is easier said than done. With cephalosporin resistance occurring at alarming rates, the FDA on 3 July 2008 proposed a withdrawal of extra-label uses of this class of antibiotics in food-producing animals, meaning farmers could no longer legally use these drugs for anything other than FDA-approved uses listed on the label. However, on 25 November 2008, the agency withdrew the proposal “in order for FDA to fully consider the comments” received from groups such as the American Association of Swine Veterinarians (AASV), which argued the ban was based on unsubstantiated data. A news item in the January/February 2009 issue of the AASV’s *Journal of Swine Health and Production* notes that “It seems . . . that given the fact that antimicrobials affect all susceptible bacteria in the animal being treated, whether or not that bacteria is on the approved label, the more rational approach would be to use an approved product for the [animal] species being treated rather than a product labeled for a different [animal] species.”

The presence of antibiotic resistance genes in surface water, groundwater, at sewage treatment plants, landfills, and a variety of agricultural and aquacultural locations means pollution of the environment has not only been chemical. And although limiting antibiotic use and creating programs that control dissemination of antibiotics can prevent the problem of resistance from worsening and may even reduce the problem, it’s unclear whether resistant strains will necessarily be replaced by susceptible ones, says Martínez. Moreover, says Gillings, “The natural disappearance of antibiotic-resistant strains is very slow—much slower than the rate of their appearance.”

Still, some studies suggest that reducing use of antibiotics at the level of an individual medical practice is associated with reduced local antibiotic resistance. For instance, in the 1 October 2007 issue of *The British Journal of General Practice*, Chris C. Butler and colleagues showed that antibiotic resistance could be effectively reduced within an observable period. Specifically, they observed an overall reduction of resistance to ampicillin (1% per year) and trimethoprim (0.6% per year) in practices that reduced their prescriptions of those drugs. Although modest, these findings may suggest the possibility of a sustained decline in resistance, wrote Butler and colleagues, thus “preserving the international reservoir of antibiotic susceptibility.”

## Figures and Tables

**Figure:**
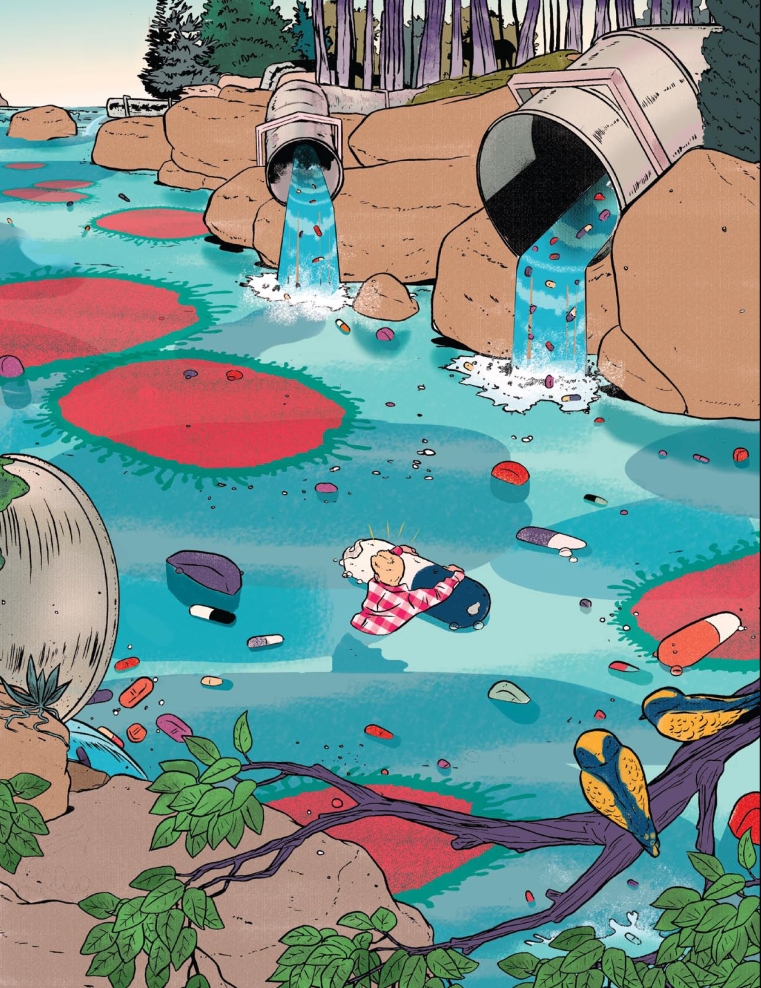


**Figure:**
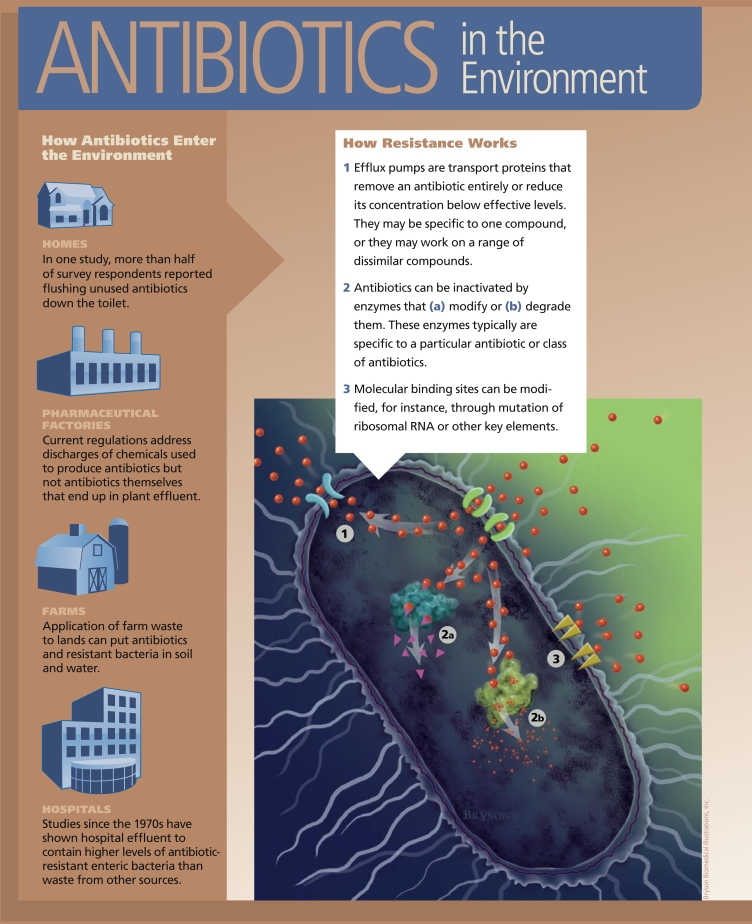


**Figure:**